# Longevity of the Negro

**Published:** 1856-02

**Authors:** 


*Longevity of the Negro.*—A letter from Rio Janeiro, in the Corrierre Mercantile, of Genoa, mentions a slave 109 years old, of the name of Francesco Tommassa Da. Sala, now living on a plantation a few miles from the capital. He was born in 1747, and had fourteen sons, who became fathers of 160 grand children, from whom sprung 70 great grand children, having, in their turn, up to the present time, produced five children, making a grand total of 249 persons, issued from one stock, still alive.—*Ibid.*

